# Uterine Rupture with Cesarean Scar Heterotopic Pregnancy with Survival of the Intrauterine Twin

**DOI:** 10.1155/2016/6832094

**Published:** 2016-12-26

**Authors:** Kimberly R. Lincenberg, Eric R. Behrman, James S. Bembry, Christine M. Kovac

**Affiliations:** ^1^Department of Obstetrics and Gynecology Residency Program, Wright State University Boonshoft School of Medicine, Dayton, OH, USA; ^2^Premier OB/GYN, Miami Valley Hospital, Dayton, OH, USA; ^3^Perinatal Partners, Miami Valley Hospital, Dayton, OH, USA

## Abstract

*Background*. Heterotopic pregnancy is a multiple gestation with both intrauterine and ectopic fetuses. A cesarean scar ectopic pregnancy is when the fetus has implanted over the previous hysterotomy site. A known complication of cesarean scar ectopic pregnancy is uterine rupture, which can cause great morbidity and mortality.* Case*. 28-year-old G5P3105 at 10 weeks with a dichorionic diamniotic gestation was found to have a ruptured uterus with expulsion of a cesarean scar ectopic pregnancy and retention of the intrauterine fetus. After uterine repair, the singleton gestation reached viability was delivered by emergent cesarean section for placental abruption.* Conclusion*. Safe management of cesarean ectopic pregnancy requires early diagnosis by ultrasonography. With early detection, management can focus on preventing maternal morbidity of uterine rupture and life-threatening hemorrhage.

## 1. Introduction

One of the rarest types of multiple gestation is a heterotopic pregnancy, which is a condition in which one fetus is intrauterine and the other is ectopic. This occurs in about 1 in 30,000 pregnancies [1]. Most commonly, the ectopic one is located in the fallopian tube. However, it can be in any other extrauterine location including a cesarean scar. Cesarean scar pregnancy is also uncommon, comprising only 6% of all ectopic pregnancies [2, 3]. Complications specific to cesarean scar ectopic pregnancy include uterine rupture in first or second trimester. The incidence of cesarean delivery has multiplied 7-fold in the last 50 years, which has in turn led to an increase in associated complications [4, 5]. Morbidity related to history of cesarean section is often from hemorrhage related to abnormal placentation or uterine rupture. These complications are normally thought of as occurring in the third trimester. We report a case of uterine rupture in the setting of cesarean scar heterotopic pregnancy in the first trimester with survival of the intrauterine twin.

## 2. Case Report

28-year-old G5P3105 at 10 weeks, 2 days with a dichorionic diamniotic gestation presented to the Emergency Department with sharp and constant abdominal pain, localized to her left lower quadrant. Review of systems was otherwise negative. The patient's medical history was noncontributory, though her obstetrical history was significant for a prior set of twins (with spontaneous conception of both sets) and a history of four low transverse cesarean sections. On physical examination, the patient's vital signs showed blood pressure 97/61, which was stable for her per chart review. Heart rate, respiratory rate, and temperature were within normal limits. Her abdomen was tender to palpation throughout, but there was no distention, guarding, or rebound. FAST (Focused Assessment with Sonography in Trauma) scan by the Emergency Medicine physician was positive for intraperitoneal fluid. Sterile speculum exam was negative for blood and the cervix appeared closed. She had hemoglobin of 8.8 g/dL and hematocrit of 22.3%, which was decreased from 12.7 g/dL and 38.8% a month earlier. Based on physical exam and ultrasound there was a strong suspicion for internal bleeding, and three units of packed red blood cells were transfused to the patient and Gynecology was consulted.

**Figure 1:**
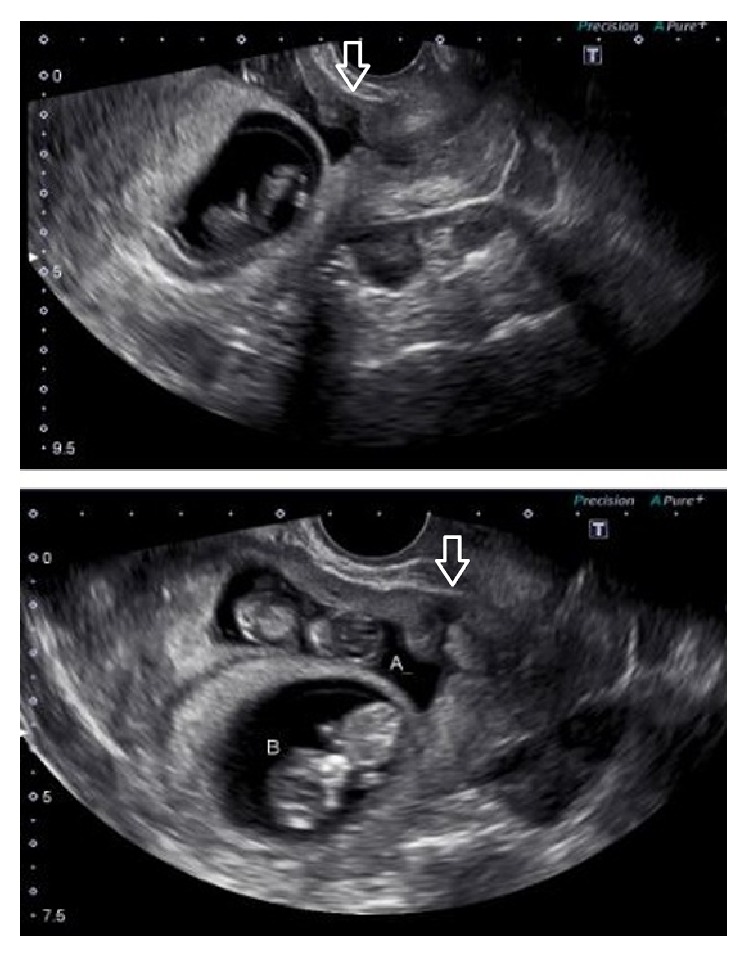
The arrows are used to demonstrate the hysterotomy scar in the lower uterine segment, into which twin A has implanted.

On presentation to the Gynecology team, the patient's vital signs were stable and her pain was well controlled with narcotic medication. An ultrasound exam was performed demonstrating two intrauterine fetuses with cardiac activity and a small subchorionic hemorrhage behind the placenta of twin B. Previous ultrasound from an outside hospital did note that the fetuses are low within the uterus (Figure 1). Due to the unilateral nature of the pain, the intraperitoneal fluid was suspected to be blood from a ruptured ovarian cyst which would hopefully resolve spontaneously. Initially we opted for conservative management in order to avoid surgical intervention during the first trimester of pregnancy, and she was admitted for observation and serial abdominal exams. By midafternoon, however, patient reported increased pain and lab work demonstrated significant decrease in hemoglobin. The decision was made to proceed with diagnostic laparoscopy for hemoperitoneum.

Prior to going to the operating room, we confirmed fetal cardiac activity of both twins by transabdominal ultrasound. The patient was placed under general anesthesia and we proceeded with a diagnostic laparoscopy. Hemoperitoneum was noted and 900 mL of blood was evacuated. Inspection of both ovaries showed bilateral simple cysts, none appeared ruptured, and no active bleeding was noted. However, upon exploration of the anterior pelvis, a fetal part was found extruding through the lower anterior uterus. We immediately converted to a laparotomy and discovered that the uterus had ruptured along the hysterotomy scar, leaving a 5 cm defect in the anterior uterus. The exteriorized fetus was removed, but chorionic tissue remained attached to the endometrium. In an attempt to save the remaining fetus, the hysterotomy was repaired with a single layer of Vicryl suture in a running locking fashion with good hemostasis noted. Another two units of packed red blood cells were transfused to the patient intraoperatively and her vital signs remained stable. Upon finishing the operation, transvaginal ultrasound was performed and confirmed the remaining intrauterine fetus with visualized cardiac activity.

The patient remained stable in the postoperative period and as her fetus was previable it was decided she could be managed with outpatient monitoring. She was counseled extensively on risks of subsequent uterine rupture with maternal or fetal demise. In addition, patient was counseled on grave prognosis of remaining fetus due to uncertainty of how well the placenta would continue to function. Ultrasounds were initially done every 2 weeks and then with increasing frequency as pregnancy continued. Possible placenta accreta as well as evidence of subchorionic hemorrhage from the area of retained chorionic tissue was demonstrated by ultrasound. The placenta was located anteriorly and extended over the lower uterine segment and hysterotomy scar. As the patient approached viability, which is 23 weeks of gestational age at our institution, the Obstetrics Department had a discussion about delivery planning. Final plan agreed upon was to perform repeat cesarean section with any indication for delivery and to prepare for possible cesarean hysterectomy due to possible placenta accreta and/or retained placenta in the hysterotomy scar.

Through the remainder of her pregnancy, she experienced multiple episodes of vaginal bleeding, requiring admissions for prolonged monitoring at 16, 20, and 21 weeks of gestational age, at which time she was admitted until delivery. The patient received a course of betamethasone for improvement of fetal lung maturity, first dose given at 22 weeks, 6 days. At 23 weeks, 1 day the patient had acute worsening of abdominal pain with vaginal bleeding of large blood clots. Decision was made to proceed with emergent cesarean section. A classical uterine incision was performed and about 300 mL of blood preceded delivery of the fetus. The placenta was delivered with the fetus. A remaining piece of placenta was removed from the anterior lower uterine segment. Banjo curettage was performed to ensure all remaining products were removed and uterus remained hemostatic with appropriate tone, so a cesarean hysterectomy was not indicated. The uterine incision was closed and good hemostasis was noted. The postoperative period was complicated by acute blood loss anemia and she required another blood transfusion; however, she recovered well and was discharged home in good condition on postoperative day three.

The fetus was born weighing 423 grams. She had APGARs of 4 at one minute, 9 at five minutes, and 9 at ten minutes. Venous blood gas (arterial blood gas sample was inadequate) showed pH of 7.29, pCO_2_ of 58.0 mEq/L, and base excess of −0.4 mmol/L. The neonate had an exceptional NICU stay; she did not develop intraventricular hemorrhage or necrotizing enterocolitis, though she did have transient retinal and renal issues that resolved by the time of discharge. She was diagnosed with respiratory distress syndrome now being managed as an outpatient with Aldactazide. She was discharged home at 41 weeks, 3 days of adjusted gestational age, weighing 2145 grams.

## 3. Discussion

Uterine rupture is a thoroughly studied risk of a pregnancy with history of previous cesarean section. The presence of a uterine scar is a strong risk factor for uterine dehiscence, which is defined as separation of the scar through the myometrium with preservation of an intact endometrial cavity. Uterine rupture implies complete opening through the endometrium, enabling expulsion of intrauterine contents and increasing risk of intraperitoneal hemorrhage [6, 7]. Along with a history of cesarean section, other risk factors for uterine rupture include cornual resection, myomectomy, and iatrogenic uterine perforation [8].

Following a low transverse cesarean section, the absolute risk of uterine rupture is 0.68%; however, the risk is increased to 1.85% after multiple cesarean sections [9]. With a history of a classical vertical incision, through the fundus of the uterus, the risk has been reported to be as high as 11.5% [9]. Incidence of cesarean delivery has increased in recent decades, from 4.5% of deliveries in 1965 to 22.6% in 1991 and up to 32.6% in 2013 [4, 5]. Increases in cesarean sections inevitably leads to increases in associated complications, including hemorrhage, blood transfusions, abnormal placentation, bowel and bladder injury, ICU admissions, and uterine rupture.

The most common management of uterine rupture is immediate delivery of a viable fetus or termination of pregnancy, with or without hysterectomy [6]. With a history of four or more cesarean sections, as in our case, there is a 1/40 chance of requiring cesarean hysterectomy, usually due to hemorrhage related to abnormal placentation [10]. In correlation to increased rates of cesarean delivery, the rate of placenta accreta increased from 1 in 30,000 in year 1950 to 1 in 533 in 2002. Of all patients who have had 4 cesarean sections, 2% will have placenta accrete [4, 8].

On literature review, most studies of uterine rupture have cases occurring in the second or third trimester, usually during labor. However, there are a few reported cases of rupture during the first trimester of pregnancy [4, 7, 8]. The first reported case was in 1982 with the extrauterine expulsion of an 11-week fetus intact in its gestational sac [4]. One remarkable case report describes a cornual rupture of a uterus containing a twin gestation at 13 weeks of gestational age. In this case there was successful repair of the defect and survival of the twins to 30 weeks [8]. Our case differs of course in the fact that we were not able to save the expelled twin.

Our patient had a heterotopic pregnancy with a cesarean scar ectopic, which was not diagnosed prior to uterine rupture. Early ultrasound noted that twin A was low in the uterus with an anterior placenta. After reviewing the ultrasound images it was noted that the uterine myometrium overlying the area of placentation of twin A was extremely thin and likely the location of the uterine scar though did not bulge as seen in some studies from our literature review. At the time of our surgery, the idea of cesarean scar heterotopic pregnancy was quite low on our differential diagnosis due to statistical unlikelihood. However, we may have considered this possibility as more likely if the ultrasound was more critically analyzed. Cesarean scar pregnancy incidence ranges from about 1 in 1800 to 2200 pregnancies and makes up only 6% of all ectopic pregnancies [2, 3]. Due to increased risks of uterine rupture and life-threatening intraperitoneal hemorrhage, cesarean scar ectopic pregnancies are managed with reduction of the pregnancy. Though there is no established protocol, acceptable methods are medical management with systemic or local methotrexate administration or surgical management with dilatation and curettage or operative laparoscopy [2]. Our patient was not a candidate for methotrexate because she was not hemodynamically stable and there was still a viable fetus.

Heterotopic pregnancy is even rarer than cesarean scar ectopic, in which a multiple gestation includes an intrauterine pregnancy and an ectopic pregnancy. Heterotopic pregnancy occurs in about 1 in 30,000 pregnancies, usually with the ectopic located in the fallopian tube [1]. There are case reports in the literature describing heterotopic cesarean scar pregnancies. In those we reviewed, the cesarean scar ectopic was reduced by aspiration or with intracardiac injection of potassium chloride, allowing for successful delivery of the normal intrauterine pregnancy [1]. In our case, heterotopic pregnancy was diagnosed after uterine rupture and loss of the fetus implanted in the uterine scar; however, we were able to repair the uterine defect and allow the intrauterine pregnancy to grow to viability and successful delivery.

## 4. Conclusion 

The rarity of heterotopic cesarean scar pregnancy is evidenced by the fact that there are limited publications detailing a protocol for appropriate management. To manage this condition in a controlled way is dependent on early diagnosis of the cesarean scar pregnancy. Our key point is the importance of ultrasound detection, so management can focus on preventing maternal morbidity of uterine rupture and life-threatening hemorrhage. Had the heterotopic gestation been diagnosed earlier, we potentially could have reduced maternal morbidity. It is crucial to remember the increased risk of uterine rupture with increasing incidence of cesarean sections. There should be suspicion of uterine rupture any time a pregnant patient with a history of cesarean section presents with abdominal pain, even in the first trimester. Ultrasonography, accompanied by clinical suspicion, is the key to diagnosis and successful management of heterotopic cesarean scar pregnancy.

## Additional Points

*Condensation.* Management of heterotopic pregnancy with cesarean scar ectopic requires early diagnosis by ultrasonography in order to prevent maternal morbidity and mortality.* Teaching Points.* (1) Ultrasound imaging in the first trimester is important in detecting cesarean scar ectopic pregnancies and thus the key to prevent associated morbidity and mortality. In particular in patients with a history of cesarean delivery, the anterior lower uterine segment should be closely evaluated by ultrasound in the first trimester. (2) Uterine rupture is associated with great morbidity and therefore should be suspected in any patient with risk factors. The risk factors include presence of uterine scar, grand multiparity, uterine distension (e.g., multiple gestation), and labor augmentation.
